# The association of neutrophil to lymphocyte ratio, platelet to lymphocyte ratio, and lymphocyte to monocyte ratio with post-thrombolysis early neurological outcomes in patients with acute ischemic stroke

**DOI:** 10.1186/s12974-021-02090-6

**Published:** 2021-02-20

**Authors:** Pengyu Gong, Yukai Liu, Yachi Gong, Gang Chen, Xiaohao Zhang, Siyu Wang, Feng Zhou, Rui Duan, Wenxiu Chen, Ting Huang, Meng Wang, Qiwen Deng, Hongchao Shi, Junshan Zhou, Teng Jiang, Yingdong Zhang

**Affiliations:** 1 Department of Neurology, Nanjing First Hospital, Nanjing Medical University, Nanjing, 210006 Jiangsu China; 2 Department of Gerontology, Nantong Third People’s Hospital, Nantong University, Nantong, 226006 Jiangsu China; 3 Department of Neurology, Haimen Hospital Affiliated to Nantong University, Nantong, 226000 Jiangsu China; 4 Department of Neurology, Jinling Hospital, Medical School of Nanjing University, Nanjing, 210000 Jiangsu China; 5 School of Basic Medicine and Clinical Pharmacy, China Pharmaceutical University, Nanjing, 210000 Jiangsu China; 6 Department of Critical Care Medicine, Nanjing First Hospital, Nanjing Medical University, Nanjing, 210006 Jiangsu China

**Keywords:** Acute ischemic stroke, Early neurological deterioration, Early neurological improvement, Intravenous thrombolysis, Neutrophil to lymphocyte ratio, Platelet to lymphocyte ratio, Lymphocyte to monocyte ratio

## Abstract

**Background and purpose:**

To investigate the association of neutrophil to lymphocyte ratio (NLR), platelet to lymphocyte ratio (PLR), and lymphocyte to monocyte ratio (LMR) with post-thrombolysis early neurological outcomes including early neurological improvement (ENI) and early neurological deterioration (END) in patients with acute ischemic stroke (AIS).

**Methods:**

AIS patients undergoing intravenous thrombolysis were enrolled from April 2016 to September 2019. Blood cell counts were sampled before thrombolysis. Post-thrombolysis END was defined as the National Institutes of Health Stroke Scale (NIHSS) score increase of ≥ 4 within 24 h after thrombolysis. Post-thrombolysis ENI was defined as NIHSS score decrease of ≥ 4 or complete recovery within 24 h. Multinomial logistic regression analysis was performed to explore the relationship of NLR, PLR, and LMR to post-thrombolysis END and ENI. We also used receiver operating characteristic curve analysis to assess the discriminative ability of three ratios in predicting END and ENI.

**Results:**

Among 1060 recruited patients, a total of 193 (18.2%) were diagnosed with END and 398 (37.5%) were diagnosed with ENI. Multinomial logistic model indicated that NLR (odds ratio [OR], 1.385; 95% confidence interval [CI] 1.238–1.551, *P* = 0.001), PLR (OR, 1.013; 95% CI 1.009–1.016, *P* = 0.001), and LMR (OR, 0.680; 95% CI 0.560–0.825, *P* = 0.001) were independent factors for post-thrombolysis END. Moreover, NLR (OR, 0.713; 95% CI 0.643–0.791, *P* = 0.001) served as an independent factor for post-thrombolysis ENI. Area under curve (AUC) of NLR, PLR, and LMR to discriminate END were 0.763, 0.703, and 0.551, respectively. AUC of NLR, PLR, and LMR to discriminate ENI were 0.695, 0.530, and 0.547, respectively.

**Conclusions:**

NLR, PLR, and LMR were associated with post-thrombolysis END. NLR and PLR may predict post-thrombolysis END. NLR was related to post-thrombolysis ENI.

**Supplementary Information:**

The online version contains supplementary material available at 10.1186/s12974-021-02090-6.

## Introduction

Stroke is one of the main reasons for mortality and morbidity at the national level in China [1, 2]. The efficacy of thrombolysis with intravenous recombinant tissue plasminogen activator was demonstrated for the patients with acute ischemic stroke (AIS) [3–5]. It has been reported that rapid recovery, which is described as early neurological improvement (ENI), can be observed in a significant proportion of AIS patients within the first 24 h after intravenous thrombolysis [3, 4]. However, there are still several patients whose symptoms would worsen and neurological deficits may aggravate within 24 h after intravenous thrombolysis, named as early neurological deterioration (END) [6–8]. Previous researches indicate that post-thrombolysis early neurological outcomes, including post-thrombolysis END as well as post-thrombolysis ENI, are relevant to the prognosis of patients treated with intravenous thrombolysis [9, 10]. Post-thrombolysis ENI can promote acceptable long-term outcomes for the patients with AIS [9], and END results in the increasing likelihood of mortality and morbidity [10]. Therefore, it is significant to explore the risk factors and the measurable biomarkers of post-thrombolysis early neurological outcomes in AIS patients.

Numerous studies have demonstrated that neuroinflammatory response plays an essential role in the pathophysiology of ischemic stroke [11–14]. Neutrophil to lymphocyte ratio (NLR), platelet to lymphocyte ratio (PLR), and lymphocyte to monocyte ratio (LMR) have recently been reported as potential novel biomarkers of baseline inflammatory process and could serve as outstanding predictors in patients with ischemic stroke [15, 16]. NLR, PLR, and LMR may have the ability to predict functional outcome in AIS patients treated with intravenous thrombolysis [17–19]. Goyal and colleagues found NLR on admission may be able to function as a prognostic biomarker of outcomes in patients with large vessel occlusion stroke [20]. What is more, previous studies showed these three novel composite inflammatory ratios, which were mentioned above, could have superior predictive capacity to those of traditional inflammatory factors [21]. Nevertheless, the relationship between these composite inflammatory ratios and post-thrombolysis early neurological outcomes in AIS patients remains uncertain and worth exploring.

In this study, we aimed to investigate the association of composite inflammatory ratios before thrombolysis, including NLR, PLR, and LMR, with post-thrombolysis END as well as ENI in AIS patients. Furthermore, we planned to explore the utility of these composite inflammatory ratios in predicting post-thrombolysis END and post-thrombolysis ENI.

## Methods

### Study design and participants

AIS patients undergoing intravenous thrombolysis within 4.5 h were recruited from the Nanjing First Hospital, Haimen Hospital Affiliated to Nantong University and Nantong Third Peoples Hospital. All the patients were treated in the stroke units and received standard treatments, for instance, antiplatelet therapy and statin therapy. Eligible participants were enrolled in the final analysis if they met the following criteria. Informed consent was obtained from participants or their legal representatives. This study was approved by the Ethics Committee of Nanjing First Hospital, Haimen Hospital Affiliated to Nantong University as well as Nantong Third Peoples Hospital.

Inclusion criteria:

1. Admission within 4.5 h after onset;
2. Treated with intravenous thrombolysis;
3. Eighteen years or older.

Exclusion criteria:

1. Severe inflammatory diseases or infectious diseases;
2. Incomplete clinical data;
3. The neurological deficits of patients cannot be evaluated over the following 24 h after admission.

### Data acquisition

On the day of admission, all the participants underwent standard assessments of demographic characteristics (age, sex, and body mass index), vascular risk factors (hypertension, diabetes mellitus, dyslipidemia, atrial fibrillation, current smoking, current drinking, previous stroke, peripheral artery disease, and coronary artery disease), medication use history (previous antiplatelet, previous anticoagulation, and previous statin), clinical assessment (stroke severity, blood pressure, hemorrhagic transformation [HT], onset to treatment time [OTT], proximal arterial occlusion [PAO], and endovascular treatment), stroke subtype, lesion location, and laboratory data. Systolic blood pressure and diastolic blood pressure were measured and recorded immediately after admission. Computed tomography, magnetic resonance, electrocardiogram, echocardiography, carotid ultrasonography, and transcranial Doppler were performed for assessing the lesion location, stroke subtype, HT, and PAO. Stroke subtype was classified according to Trial of Org 10172 in Acute Stroke Treatment (TOAST) criteria [22]. Laboratory data included total cholesterol (TC), triglyceride (TG), high-density lipoprotein (HDL), low-density lipoprotein (LDL), fasting blood glucose (FBG), hypersensitive C-reactive protein (Hs-CRP), NLR, PLR, and LMR.

### Measurement of composite inflammatory ratios from blood cell counts

Blood cell counts, including total leukocyte counts, neutrophil counts, lymphocyte counts, monocyte counts, and platelet counts, were sampled from each participant in emergency room on admission before intravenous thrombolysis. Then, the cell counts were analyzed by an auto-analyzer (XE-2100, Sysmex, Kobe, Japan) and utilized to calculate composite inflammatory ratios (including NLR, PLR, and LMR). NLR was calculated as neutrophil counts/lymphocyte counts. PLR was calculated as platelet counts/lymphocyte counts. LMR was calculated as lymphocyte counts/monocyte counts.

### Definition of post-thrombolysis early neurological deterioration and early neurological improvement

Stroke severity was assessed using NIHSS score on the day of admission and continued 2–3 times over the following 24 h after admission by two certified neurologists. All the certified neurologists in the three centers underwent unified training for NIHSS score evaluation and were blind to our study. In case of disagreement about the NIHSS score evaluation, a third neurologist in this center was invited for a final decision. Post-thrombolysis END was defined as an increase in the NIHSS score by ≥ 4 points in the total score within 24 h after thrombolysis [7, 23, 24]. Meanwhile, post-thrombolysis ENI was defined as a decrease in the NIHSS score by ≥ 4 points in the total score or a complete resolution of neurological deficits within 24 h after thrombolysis [25–27].

### Statistical analysis

Statistical analyses were performed using R version 4.0.3 software (http://www.R-project.org/). All participants were categorized into 3 groups according to post-thrombolysis early neurological outcomes (END group, neither END nor ENI group as well as ENI group). Categorical variables were expressed as *n* (%), and continuous variables were expressed as means (standard deviation, SD) or medians (interquartile range, IQR). Differences in baseline characteristics between groups were analyzed using one-way ANOVA test or Mann-Whitney *U* test for continuous variables as well as the chi-squared test or Fisher’s exact test for categorical variables, as appropriate. We used the violin plots to show the distribution of NLR, PLR, and LMR among the END group, neither the END nor ENI group as well as the ENI group. We also used crude model analyses to detect the risk factors of END and ENI. By setting “neither END nor ENI” as the reference category, multinomial logistic regression analysis was adjusted for all potential confounders with statistically significant association at *P* < 0.05 in comparison of baseline demographics and clinical characteristics among three groups according to early neurological outcomes. A MedCalc 15.6.0 (MedCalc Software Acacialaan 22, B-8400 Ostend, Belgium) packet program was used to obtain receiver operating characteristic (ROC) curve to test the overall discriminative ability of NLR, PLR, and LMR for post-thrombolysis END as well as post-thrombolysis ENI. Youden Index was calculated as the Sensitivity+Specificity–1. A two-tailed value of *P* < 0.05 was considered significant.

## Results

From April 2016 to September 2019, a total of 1235 AIS patients treated with intravenous thrombolysis were screened for 24 h in this study (Additional file 1: Figure S1). Ninety-two patients’ neurological deficits could not be evaluated over the following 24 h after admission. Meanwhile, eighty-three patients were excluded for the following reasons: nineteen patients were excluded for severe inflammatory or infectious diseases, and sixty-four patients were excluded for incomplete data. A total of 1060 subjects were included for the final analysis.

**Fig. 1:**
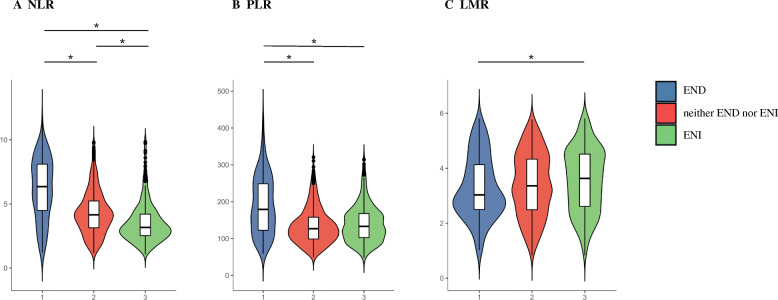
The violin plot in distribution of composite inflammatory ratios (neutrophil to lymphocyte ratio, platelet to lymphocyte ratio, and lymphocyte to monocyte ratio) among the different early neurological outcome group. **a** The violin plot in distribution of neutrophil-lymphocyte ratio among the different early neurological outcome group. **b** The violin plot in distribution of platelet-lymphocyte ratio among the different early neurological outcome group. **c** The violin plot in distribution of lymphocyte-monocyte ratio among the different early neurological outcome group

After admission, post-thrombolysis END was observed in 193 patients (18.2%), and ENI was observed in 398 patients (37.5%), respectively. Baseline characteristics of the study participants according to post-thrombolysis early neurological outcomes (END group, neither the END nor ENI group as well as the ENI group) are provided in Table 1. Significant differences among the three group are described as follows: age (*P* = 0.001), previous antiplatelet (*P* = 0.002), NIHSS (*P* = 0.001), diastolic blood pressure (*P* = 0.034), HT (*P* = 0.001), OTT (*P* = 0.001), PAO (*P* = 0.001), stroke subtype (*P* = 0.001), FBG (*P* = 0.001), Hs-CRP (*P* = 0.001), NLR (*P* = 0.001), PLR (*P* = 0.001), and LMR (*P* = 0.014). Figure 1 showed the violin plots of NLR, PLR, and LMR among three groups. The patients in the END group had elevated levels of NLR and PLR than those patients in the neither END nor ENI group. Compared to the patients in the ENI group, the patients in the END group possessed higher levels of NLR, PLR, and lower levels of LMR. What is more, the patients in the neither the END nor ENI group owned upgraded levels of NLR than the patients in the ENI group (all *P* < 0.05).

**Table 1:** Demographics and clinical characteristics of the subgroup according to early neurological outcomes

Variable	END group (*n* = 193)	Neither END nor ENI group (*n* = 469)	ENI group (*n* = 398)	*P*
Demographic characteristics				
Age, years	73.2 ± 11.5	69.6 ± 12.0 ^a^	68.1 ± 12.1 ^b^	0.001
Male, *n* (%)	121 (62.7)	319 (68.0)	260 (65.3)	0.303
BMI, kg/m^2^	24.4 ± 3.6	24.1 ± 3.5	23.9 ± 3.6	0.242
Vascular risk factors, *n* (%)				
Hypertension	132 (68.4)	315 (67.2)	275 (69.1)	0.816
Diabetes mellitus	51 (26.4)	116 (24.7)	77 (19.3) ^b^	0.079
Dyslipidemia	51 (26.4)	120 (25.6)	114 (28.6)	0.592
Atrial fibrillation	49 (25.4)	96 (20.5)	75 (18.8)	0.180
Current smoking	59 (30.6)	149 (31.8)	129 (32.4)	0.903
Current drinking	69 (35.8)	145 (30.9)	129 (32.4)	0.482
Previous stroke	38 (19.7)	114 (24.3)	80 (20.1)	0.238
Peripheral artery disease	11 (5.7)	30 (6.4)	30 (7.5)	0.662
Coronary artery disease	38 (19.7)	83 (17.7)	81 (20.4)	0.603
Medication use history, *n* (%)				
Previous antiplatelet	21 (10.9)	88 (18.8) ^a^	91 (22.9) ^b^	0.002
Previous statin	15 (7.8)	49 (10.4)	42 (10.6)	0.521
Previous anticoagulation	5 (2.6)	11 (2.3)	5 (1.3)	0.391
Clinical assessment				
NIHSS, score	11 (5, 17)	5 (3, 12) ^a^	8 (4, 14) ^b,c^	0.001
SBP, mmHg	146.9 ± 23.6	151.8 ± 25.7	148.3 ± 22.6	0.171
DBP, mmHg	87.8 ± 16.2	89.4 ± 14.4	86.8 ± 14.6 ^c^	0.034
HT, *n* (%)	32 (16.6)	37 (7.9) ^a^	13 (3.3) ^b,c^	0.001
OTT, minute	165.00 (120.00, 200.00)	160.00 (115.00, 200.00)	130.00 (90.00, 180.00) ^b,c^	0.001
PAO, *n* (%)	77 (39.9)	134 (28.6) ^a^	91 (22.9) ^b^	0.001
Endovascular treatment, *n* (%)	43 (22.3)	77 (16.4)	65 (16.3)	0.148
Stroke subtype, *n* (%)				0.001
LAA	73 (37.8)	169 (36.0)	147 (36.9)	
CE	62 (32.1)	120 (25.6)	132 (33.2)	
SAO	27 (14.0)	138 (29.4) ^a^	83 (20.9)	
SOE	8 (4.1)	16 (3.4)	8 (2.0)	
SUE	23 (12.0)	26 (5.6) ^a^	28 (7.0)	
Lesion location, *n* (%)				0.616
Anterior circulation	149 (77.2)	357 (76.1)	294 (73.9)	
Posterior circulation	44 (22.8)	112 (23.9)	104 (26.1)	
Laboratory data				
TC, mmol/L	4.41 ± 1.11	4.38 ± 1.11	4.32 ± 1.10	0.591
TG, mmol/L	1.14 (0.82, 1.68)	1.19 (0.86, 1.70)	1.20 (0.85, 1.75)	0.744
HDL, mmol/L	1.21 ± 0.68	1.14 ± 0.36	1.13 ± 0.49	0.214
LDL, mmol/L	2.53 (1.95, 3.20)	2.63 (1.98, 3.23)	2.59 (1.96, 3.27)	0.733
FBG, mmol/L	7.30 ± 2.95	6.28 ± 2.47 ^a^	6.86 ± 3.19 ^c^	0.001
Hs-CRP, mg/L	7.25 (2.66, 14.55)	4.28 (2.11, 7.55) ^a^	4.85 (2.16, 8.61) ^b^	0.001
NLR	6.09 (4.43, 8.02)	4.15 (3.14, 5.26) ^a^	3.17 (2.54, 4.22) ^b,c^	0.001
PLR	179.1 (122.1, 251.1)	126.7 (98.8, 158.0) ^a^	133.1 (102.5, 168.2) ^b^	0.001
LMR	3.03 (2.50, 4.13)	3.36 (2.48, 4.34)	3.63 (2.61, 4.52) ^b^	0.014

*Abbreviations*: *END* early neurological deterioration, *ENI*, early neurological improvement, *BMI* body mass index, *NIHSS* National Institute of Health Stroke Scale, *SBP* systolic blood pressure, *DBP* diastolic blood pressure, *HT* hemorrhagic transformation, *OTT* onset to treatment time, *PAO* proximal arterial occlusion, *LAA* large-artery atherosclerosis, *CE* cardioembolism, *SAO* small-artery occlusion, *SOE* stroke of other determined etiology, SUE stroke of undetermined etiology, *TC* total cholesterol, *TG* triglyceride, *LDL* low-density lipoprotein, *HDL* high-density lipoprotein, *FBG* fasting blood glucose, *Hs-CRP* hyper-sensitive C-reactive protein, *NLR* neutrophil-lymphocyte ratio, *PLR* platelet-lymphocyte ratio, *LMR* lymphocyte-monocyte ratio

^a^This variable is different significantly between END group and neither END nor ENI group, *P* < 0.05

^b^This variable is different significantly between ENI group and END group, *P* < 0.05

^c^This variable is different significantly between neither END nor ENI group and ENI group, *P* < 0.05

Table 2 illustrated the results of crude models for post-thrombolysis END and post-thrombolysis ENI. Crude models for END showed that age, previous antiplatelet, NIHSS, HT, OTT, PAO, stroke subtype, FBG, Hs-CRP, NLR, PLR, and LMR might be associated with post-thrombolysis END (*P* < 0.05). Furthermore, crude models also exhibited that age, diabetes mellitus, previous antiplatelet, diastolic blood pressure, HT, OTT, PAO, NLR, PLR, and LMR might be related to post-thrombolysis ENI (*P* < 0.05).

**Table 2:** Univariate Logistic regression analysis for risk factors with post-thrombolysis END and post-thrombolysis ENI

Variable	Crude model for post-thrombolysis END		Crude model for post-thrombolysis ENI	
	OR (95%CI)	*P*	OR (95%CI)	*P*
Demographic characteristics				
Age	1.033 (1.018–1.048)	0.001	0.982 (0.972–0.993)	0.002
Male	0.813 (0.587–1.125)	0.211	0.950 (0.729–1.237)	0.702
BMI	1.032 (0.987–1.079)	0.169	0.974 (0.940–1.010)	0.159
Vascular risk factors				
Hypertension	1.033 (0.737–1.447)	0.851	1.070 (0.819–1.399)	0.629
Diabetes mellitus	1.254 (0.877–1.794)	0.215	0.711 (0.524–0.964)	0.028
Dyslipidemia	0.972 (0.682–1.383)	0.873	1.153 (0.872–1.523)	0.317
Atrial fibrillation	1.385 (0.962–1.995)	0.080	0.828 (0.606–1.130)	0.235
Current smoking	0.933 (0.665–1.308)	0.687	1.047 (0.802–1.366)	0.737
Current drinking	1.204 (0.868–1.671)	0.226	1.004 (0.770–1.309)	0.977
Previous stroke	0.847 (0.574–1.249)	0.402	0.848 (0.625–1.150)	0.288
Peripheral artery disease	0.813 (0.419–1.577)	0.540	1.235 (0.758–2.012)	0.397
Coronary artery disease	1.049 (0.708–1.555)	0.810	1.140 (0.833–1.561)	0.412
Medication use history				
Previous antiplatelet	0.469 (0.290–0.760)	0.002	1.504 (1.102–2.053)	0.010
Previous anticoagulation	1.415 (0.512–3.909)	0.504	0.514 (0.187–1.413)	0.197
Previous statin	0.848 (0.638–1.127)	0.256	1.050 (0.855–1.289)	0.642
Clinical assessment				
NIHSS	1.051 (1.030–1.072)	0.001	1.007 (0.990–1.024)	0.446
SBP	0.995 (0.989–1.002)	0.195	0.998 (0.993–1.002)	0.359
DBP	0.998 (0.993–1.009)	0.737	0.990 (0.982–0.999)	0.023
HT	3.248 (2.020–5.221)	0.001	0.290 (0.158–0.532)	0.001
OTT	1.004 (1.001–1.006)	0.011	0.993 (0.990–0.995)	0.001
PAO	1.894 (1.368–2.623)	0.001	0.634 (0.476–0.843)	0.002
Endovascular treatment	1.464 (0.997–2.148)	0.052	0.882 (0.633–1.228)	0.456
Stroke subtype				
LAA	Reference		Reference	
CE	1.065 (0.731–1.552)	0.743	1.194 (0.881–1.618)	0.252
SAO	0.529 (0.329–0.849)	0.008	0.828 (0.592–1.157)	0.268
SOE	1.443 (0.623–3.341)	0.392	0.549 (0.240–1.253)	0.154
SUE	1.844 (1.063–3.197)	0.029	0.941 (0.566–1.563)	0.813
Lesion location				
Anterior circulation	Reference		Reference	
Posterior circulation	0.890 (0.615–1.288)	0.537	1.147 (0.861–1.528)	0.347
Laboratory data				
TC	1.003 (0.996–1.010)	0.447	0.999 (0.994–1.003)	0.591
TG	1.000 (0.998–1.002)	0.623	1.001 (0.996–1.005)	0.655
HDL	1.255 (0.953–1.654)	0.106	0.889 (0.664–1.190)	0.428
LDL	0.999 (0.992–1.005)	0.708	1.002 (0.998–1.005)	0.415
FBG	1.084 (1.032–1.138)	0.002	1.035 (0.991–1.080)	0.119
Hs-CRP	1.033 (1.018–1.048)	0.001	0.997 (0.984–1.010)	0.630
NLR	1.661 (1.528–1.807)	0.001	0.678 (0.626–0.735)	0.001
PLR	1.014 (1.011–1.017)	0.001	0.997 (0.995–0.999)	0.004
LMR	0.869 (0.758–0.996)	0.043	1.152 (1.033–1.284)	0.011

*Abbreviation*: *END* early neurological deterioration, *ENI* early neurological improvement, *OR* odds ratio, *CI* confidence interval, *BMI* body mass index, *NIHS*S National Institute of Health Stroke Scale, *SBP* systolic blood pressure, *DBP* diastolic blood pressure, *HT* hemorrhagic transformation, *OTT* onset to treatment time, *PAO* proximal arterial occlusion, *LAA* large-artery atherosclerosis, *CE* cardioembolism, *SAO* small-artery occlusion, *SOE* stroke of other determined etiology, *SUE,* stroke of undetermined etiology, *TC* total cholesterol, *TG* triglyceride, *LDL* low-density lipoprotein*, HDL* high-density lipoprotein, *FBG* fasting blood glucose, *Hs-CRP* hyper-sensitive C-reactive protein, *NLR* neutrophil-lymphocyte ratio, *PLR* platelet-lymphocyte ratio, *LMR* lymphocyte-monocyte ratio

Table 3 displayed the results of the multiple logistic regression model for post-thrombolysis END and post-thrombolysis ENI. After adjustment for all potential confounders, NLR (odds ratio [OR], 1.385; 95% confidence interval [CI] 1.238–1.551, *P* = 0.001), PLR (OR, 1.013; 95% CI 1.009–1.016, *P* = 0.001), and LMR (OR, 0.680; 95% CI 0.560–0.825, *P* = 0.001) were identified as independent factors for post-thrombolysis END. What is more, NLR (OR, 0.713; 95% CI 0.643–0.791, *P* = 0.001) remained an independent factor for post-thrombolysis ENI.

**Table 3:** Multinomial logistic regression models for post-thrombolysis END and post-thrombolysis ENI

Variable	Post-thrombolysis END		Post-thrombolysis ENI	
	OR (95%CI)	*P*	OR (95%CI)	*P*
Age	1.015 (0.997–1.034)	0.109	0.980 (0.967–0.992)	0.002
Previous antiplatelet	0.654 (0.363–1.179)	0.158	1.249 (0.865–1.804)	0.235
NIHSS	1.041 (1.012–1.070)	0.005	1.029 (0.997–1.055)	0.102
DBP	0.997 (0.984–1.011)	0.704	0.985 (0.974–0.995)	0.003
HT	1.637 (0.849–3.157)	0.142	0.456 (0.229–0.908)	0.025
OTT	1.001 (0.998–1.005)	0.504	0.992 (0.990–0.995)	0.001
PAO	1.277 (0.834–1.954)	0.260	0.688 (0.489–0.968)	0.032
Stroke subtype				
LAA	Reference		Reference	
CE	0.988 (0.287–3.402)	0.984	0.365 (0.123–1.080)	0.069
SAO	0.263 (0.114–0.608)	0.002	0.649 (0.332–1.269)	0.206
SOE	0.477 (0.222–1.023)	0.057	1.229 (0.640–2.360)	0.535
SUE	0.410 (0.193–0.872)	0.021	0.862 (0.454–1.638)	0.651
FBG	1.112 (1.038–1.191)	0.003	1.054 (0.996–1.116)	0.070
Hs-CRP	1.036 (1.015–1.057)	0.002	1.013 (0.995–1.032)	0.156
NLR	1.385 (1.238–1.551)	0.001	0.713 (0.643–0.791)	0.001
PLR	1.013 (1.009–1.016)	0.001	1.005 (0.999–1.011)	0.084
LMR	0.680 (0.560–0.825)	0.001	1.023 (0.889–1.172)	0.751

*Abbreviations*: *END* early neurological deterioration, *ENI* early neurological improvement, *OR* odds ratio, *CI* confidence interval, *NIHSS* National Institute of Health Stroke Scale, *DBP* diastolic blood pressure, *HT* hemorrhagic transformation, *OTT* onset to treatment time, *PAO* proximal arterial occlusion, *LAA* large-artery atherosclerosis, *CE* cardioembolism, *SA*O small-artery occlusion, *SOE* stroke of other determined etiology, *SUE* stroke of undetermined etiology, *FBG* fasting blood glucose, *Hs-CRP* hyper-sensitive C-reactive protein, *NLR* neutrophil-lymphocyte ratio, *PLR* platelet-lymphocyte ratio, *LMR* lymphocyte-monocyte ratio

**Fig. 2:**
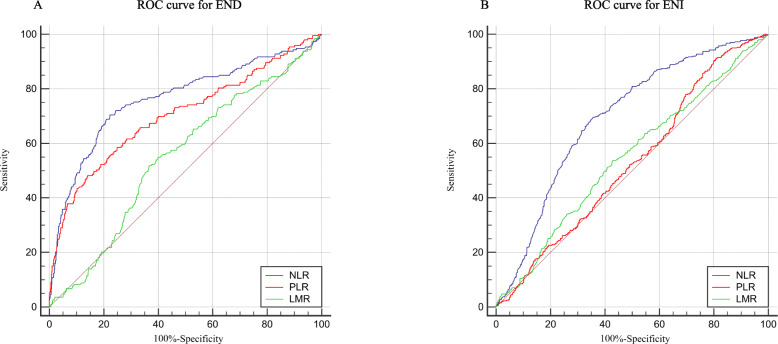
Receiver operating characteristic curve for composite inflammatory ratios (neutrophil to lymphocyte ratio, platelet to lymphocyte ratio, and lymphocyte to monocyte ratio) to predict post-thrombolysis early neurological outcome. **a** Receiver operating characteristic curve to predict post-thrombolysis early neurological deterioration. **b** Receiver operating characteristic curve to predict post-thrombolysis early neurological improvement

ROC curves, which were depicted in Fig. 2, were used to test the overall discriminative ability of these three composite inflammatory ratios for outcomes. We observed that the area under curve (AUC) of NLR, PLR, and LMR to discriminate post-thrombolysis END were 0.763 (95% CI, 0.736–0.788), 0.703 (95% CI, 0.675–0.730), and 0.551 (95% CI, 0.521–0.581) (Fig. 2a). To predict post-thrombolysis END, AUC of NLR was superior to PLR (0.763 versus 0.703, *P* = 0.010) and LMR (0.763 versus 0.551, *P* = 0.001). Moreover, AUC of PLR was superior to LMR (0.703 versus 0.551, *P* = 0.001). Meanwhile, the AUC of NLR, PLR, and LMR to discriminate post-thrombolysis ENI were 0.695 (95% CI, 0.666–0.722), 0.530 (95% CI, 0.499–0.560), and 0.547 (95% CI, 0.516–0.577) (Fig. 2b). To predict post-thrombolysis ENI, AUC of NLR was superior to PLR (0.695 versus 0.530, *P* = 0.001) and LMR (0.695 versus 0.547, *P* = 0.001). However, there was no significant difference between the AUC of PLR and LMR (0.530 versus 0.547, *P* = 0.461). We also established optimal cutoff values at which the Youden index was highest. The details of optimal cutoff values for NLR, PLR, and LMR as predictors of post-thrombolysis END and ENI were described in Additional file 2: Table S1.

## Discussion

To our knowledge, this is the first study with relatively large samples to investigate the association between composite inflammatory ratios before thrombolysis and post-thrombolysis early neurological outcomes. Our study showed that the prevalence of post-thrombolysis END and post-thrombolysis ENI were 18.2% as well as 37.5%, respectively. This prevalence is in line with the previous researches [24–28]. In this observational study, we found NLR, PLR, and LMR were associated with post-thrombolysis END. Moreover, NLR was related to post-thrombolysis ENI. In general, a biomarker with 0.7 < AUC < 0.9 indicates a moderate diagnostic value. Therefore, NLR and PLR may be capable of predicting post-thrombolysis END.

During recent years, neuroinflammation has drawn more and more attention, and numerous studies have confirmed that inflammatory mechanisms play crucial roles in the pathogenesis and progression of ischemic stroke [29–34]. Peripheral leukocytes are guided by the inflammatory cytokines and chemokines, which are released from ischemic tissues [35]. Conversely, peripheral leukocytes may affect ischemic tissues as well [35]. Lymphocyte counts have been considered to have neuroprotective effect and contribute to neurological function improvement [35]. Both peripheral monocytes and neutrophils could perform as the source of matrix metalloproteinase-9, which would lead to HT and symptomatic deterioration [7, 36, 37]. Furthermore, it is reported that neutrophils can induce free oxygen radicals and cause brain injury [38]. Meanwhile, AIS may result in platelet function abnormality, and excessive activation and accumulation of platelets could hamper stroke recovery [39]. NLR, PLR, and LMR are three composite ratios in the combination of different inflammatory parameters, hence they may be able to provide more information about immunological activities during the pathogenesis of ischemic stroke. What is more, these three composite inflammatory ratios can be calculated from blood cell counts, so they are relatively accessible. Previous studies exhibited that NLR, PLR, and LMR could predict the clinical outcome in AIS patients [16, 18, 40]. NLR and LMR can also foretell HT after ischemic stroke [41, 42]. Moreover, high NLR and PLR might be associated with symptomatic internal carotid artery stenosis [43]. Nam and colleagues found that upgraded NLR may portend stroke-associated pneumonia [44]. In addition, elevated levels of PLR are associated with post-stroke depression according to the study of Huang et al. [45]. The findings of our study have supplemented the roles of NLR, PLR, and LMR in cerebrovascular disease and provide new ideas for clinical practice, too.

In this study, we also discovered that small artery occlusion subtype, stroke of undetermined etiology subtype (both take the large-artery atherosclerosis subtype as the reference category), baseline NIHSS, FBG, and Hs-CRP were related to post-thrombolysis END. In addition, multiple logistic regression models showed that age, diastolic blood pressure, HT, OTT, and PAO were connected with post-thrombolysis ENI. Previous researches confirmed that age, elevated levels of baseline NIHSS, OTT, and FBG may be able to be the risk factors of neurological recovery [7, 8, 46]. Moreover, HT could serve as one reason for END [6, 47], and PAO may lead to symptoms worsening and poor prognosis in AIS patients [48]. Hu et al. found that diastolic blood pressure might be associated with END [49]. One current study discovered that TOAST type of large-artery atherosclerosis was one of independent predictors for END [50], which hinted that the patients with other subtypes might be less likely to develop END than those patients with large-artery atherosclerosis subtype. However, the mechanisms of stroke subtype in the post-thrombolysis END still remain uncertain, and future studies are warranted to figure out the relationship between TOAST subtype and early neurological outcomes. Serum Hs-CRP could also function as an inflammatory biomarker. Our previous study showed that Hs-CRP could predict progressive motor deficits, a subtype of END, in patients with penetrating artery infarctions [51]. Moreover, there were other studies that found serum Hs-CRP levels might be independently relevant with END, after adjustment for confounders [7, 52].

Our study has several potential limitations. Firstly, all the participants enrolled were Chinese patients treated with intravenous thrombolysis, so the associations between composite inflammatory ratios before thrombolysis and post-thrombolysis early neurological outcomes need to be tested in non-Chinese populations. Second, some risk factors that may be linked to END or ENI, such as serum homocysteine [53] and trimethylamine N-oxide levels [54], were unavailable in this study. These biomarkers might serve as potential independent predictors for END. Therefore, we attempt to collect these variables prospectively. Third, it is reported that composite inflammatory ratios might be able to vary significantly during hospitalization. Our future research needs dynamic examination of composite inflammatory ratios, not only composite inflammatory ratios before thrombolysis. Moreover, there were more than one neurologist that assessed stroke severity, which would lead to bias on evaluation of NIHSS, even though these neurologists have undergone standardized training. In addition, machine learning, which could use algorithms based on statistical assumptions and mathematical rules to learn patterns [55], is a novel approach to make classifications for the most probable reason. We aim to apply machine learning approach to predict the neurological outcomes in the future study. Finally, we have not developed an independent validation cohort, which is able to provide more credibility. Therefore, it is meaningful and advantageous for us to establish an independent validation cohort in the coming research. Despite these limitations mentioned above, it is the first time to explore the relationship between composite inflammatory ratios before thrombolysis and post-thrombolysis early neurological outcomes, including END and ENI, with relatively large samples.

## Conclusion

In summary, our study showed that NLR before thrombolysis, LMR before thrombolysis and PLR before thrombolysis were associated with post-thrombolysis END. Meanwhile, NLR before thrombolysis was related to post-thrombolysis ENI. What is more, NLR and PLR may have the ability to predict post-thrombolysis END. NLR, PLR, and LMR, which are easily available, might have utility as an inclusion criterion for future clinical trials about thrombolysis. Further investigations will be required to verify these results about post-thrombolysis early neurological outcomes.

## Supplementary Information

**Additional file 1: Figure S1.** The flowchart of participants selection.

**Additional file 2: Table S1.** The ROC curves for post-thrombolysis END and post-thrombolysis ENI.

**Additional file 3: Table S2.** The ROC curves for post-thrombolysis END.

**Additional file 4: Table S3.** The ROC curves for post-thrombolysis ENI.

## Data Availability

The data that support the findings of this study are available from the corresponding author upon reasonable request.

## Abbreviations

AIS: Acute ischemic stroke
ENI: Early neurological improvement
END: Early neurological deterioration
NLR: Neutrophil to lymphocyte ratio
PLR: Platelet to lymphocyte ratio
LMR: Lymphocyte to monocyte ratio
HT: Hemorrhagic transformation
OTT: Onset to treatment time
PAO: Proximal arterial occlusion
TOAST: Trial of Org 10172 in Acute Stroke Treatment
TC: Total cholesterol
TG: Triglyceride
HDL: High-density lipoprotein
LDL: Low-density lipoprotein
FBG: Fasting blood glucose
Hs-CRP: Hypersensitive C-reactive protein
OR: Odds ratio
95% CI: 95% Confidence interval
AUC: Area under the curve
