# Supplementary figures and images for: The association of neutrophil to lymphocyte ratio, platelet to lymphocyte ratio, and lymphocyte to monocyte ratio with post-thrombolysis early neurological outcomes in patients with acute ischemic stroke
Source: J Neuroinflammation. 2021 Feb 20;18:51. doi: 10.1186/s12974-021-02090-6 (PMC7896410; doi:10.1186/s12974-021-02090-6)

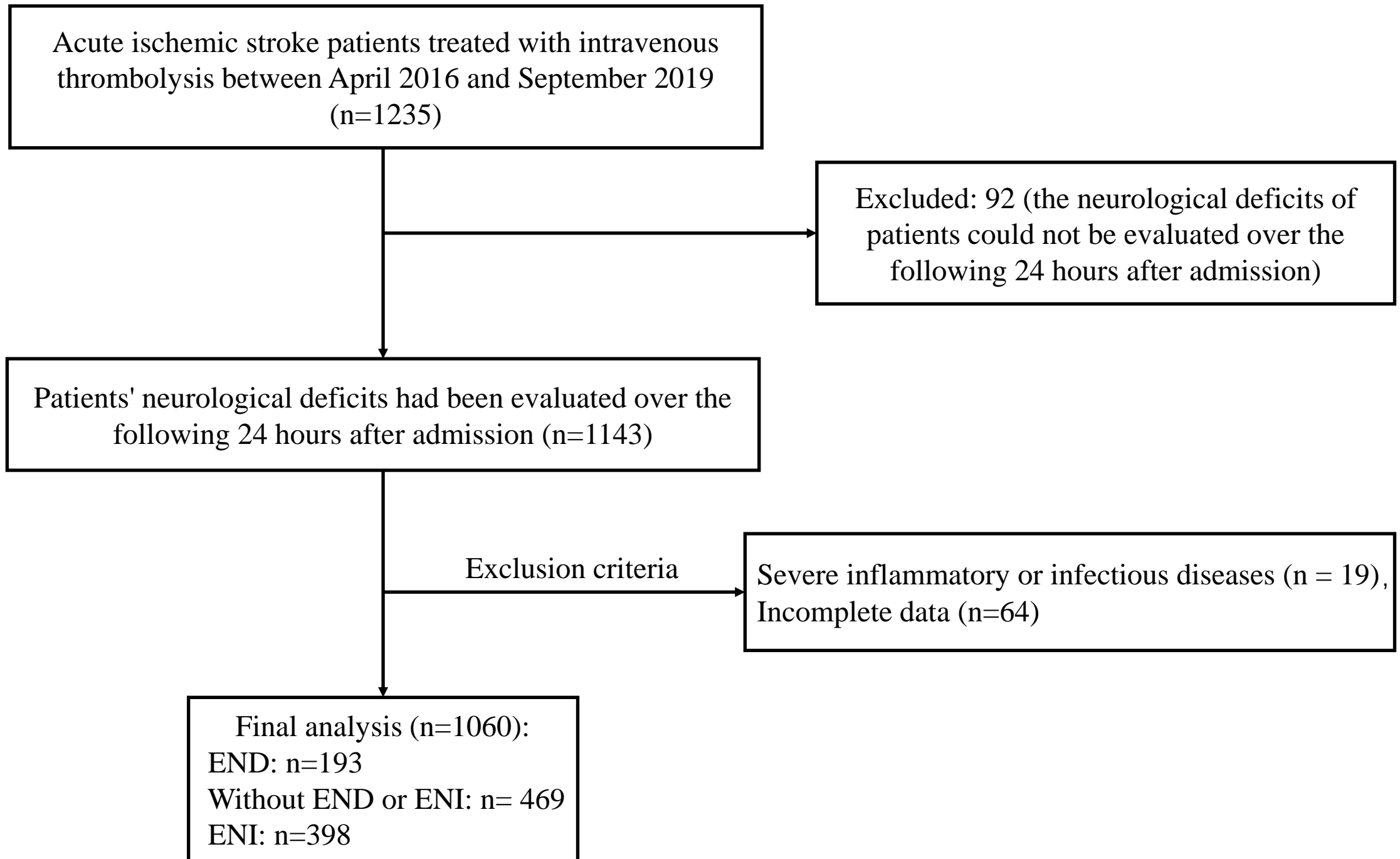

Supplement: Supplementary file 1 — Additional file 1: Figure S1. The flowchart of participants selection. [file 12974_2021_2090_MOESM1_ESM.pdf]
