# Supplementary material for: The association of neutrophil to lymphocyte ratio, platelet to lymphocyte ratio, and lymphocyte to monocyte ratio with post-thrombolysis early neurological outcomes in patients with acute ischemic stroke
Source: J Neuroinflammation. 2021 Feb 20;18:51. doi: 10.1186/s12974-021-02090-6 (PMC7896410; doi:10.1186/s12974-021-02090-6)
Supplement: Supplementary file 2 — Additional file 2: Table S1. The ROC curves for post-thrombolysis END and post-thrombolysis ENI. [file 12974_2021_2090_MOESM2_ESM.docx]

Additional file 2: Table S1. ROC curves for post-thrombolysis END and post-thrombolysis ENI

|  | AUC | 95% CI | optimal cutoff value | specificity | sensitivity | Youden index |
| --- | --- | --- | --- | --- | --- | --- |
|  | ROC curves for post-thrombolysis END | | | | | |
| NLR | 0.763 | 0.736-0.788 | 4.92 | 77.74 | 70.47 | 0.482 |
| PLR | 0.703 | 0.675-0.730 | 188.46 | 85.93 | 48.19 | 0.341 |
| LMR | 0.551 | 0.521-0.581 | 3.04 | 63.32 | 51.81 | 0.151 |
|  | ROC curves for post-thrombolysis ENI | | | | | |
| NLR | 0.695 | 0.666-0.722 | 3.89 | 64.35 | 69.60 | 0.340 |
| PLR | 0.530 | 0.499-0.560 | 201.28 | 20.09 | 90.20 | 0.103 |
| LMR | 0.547 | 0.516-0.577 | 3.52 | 57.10 | 53.77 | 0.109 |

Abbreviation: ROC, receiver operating characteristic; END, early neurological deterioration; ENI, early neurological improvement; AUC, area under curve; CI, confidence interval; NLR, neutrophil-lymphocyte ratio; PLR. platelet-lymphocyte ratio; LMR, lymphocyte-monocyte ratio.
