# Supplementary material for: The association of neutrophil to lymphocyte ratio, platelet to lymphocyte ratio, and lymphocyte to monocyte ratio with post-thrombolysis early neurological outcomes in patients with acute ischemic stroke
Source: J Neuroinflammation. 2021 Feb 20;18:51. doi: 10.1186/s12974-021-02090-6 (PMC7896410; doi:10.1186/s12974-021-02090-6)
Supplement: Supplementary file 3 — Additional file 3: Table S2. The ROC curves for post-thrombolysis END. [file 12974_2021_2090_MOESM3_ESM.docx]

| Additional file 3: Table S2. The ROC curves for post-thrombolysis END | | |
| --- | --- | --- |
| variable | Area under curve | 95% CI |
| NLR | 0.763 | 0.736-0.788 |
| PLR | 0.703 | 0.675-0.730 |
| LMR | 0.551 | 0.521-0.581 |
| Age | 0.594 | 0.564-0.624 |
| NIHSS | 0.611 | 0.581-0.640 |
| OTT | 0.563 | 0.533-0.593 |
| FBG | 0.591 | 0.560-0.621 |
| Hs-CRP | 0.613 | 0.582-0.642 |

Abbreviation: ROC, receiver operating characteristic; END, early neurological deterioration; AUC, area under curve; CI, confidence interval; NLR, neutrophil-lymphocyte ratio; PLR. platelet-lymphocyte ratio; LMR, lymphocyte-monocyte ratio; NIHSS, national institute of health stroke scale; OTT, onset to treatment time; FBG, fasting blood glucose; Hs-CRP, hyper-sensitive c-reactive protein.
