# Supplementary material for: The association of neutrophil to lymphocyte ratio, platelet to lymphocyte ratio, and lymphocyte to monocyte ratio with post-thrombolysis early neurological outcomes in patients with acute ischemic stroke
Source: J Neuroinflammation. 2021 Feb 20;18:51. doi: 10.1186/s12974-021-02090-6 (PMC7896410; doi:10.1186/s12974-021-02090-6)
Supplement: Supplementary file 4 — Additional file 4: Table S3. The ROC curves for post-thrombolysis ENI. [file 12974_2021_2090_MOESM4_ESM.docx]

| Additional file 4: Table S3. The ROC curves for post-thrombolysis ENI | | |
| --- | --- | --- |
| variable | Area under curve | 95% CI |
| NLR | 0.695 | 0.666-0.722 |
| PLR | 0.530 | 0.499-0.560 |
| LMR | 0.547 | 0.516-0.577 |
| Age | 0.561 | 0.530-0.591 |
| OTT | 0.615 | 0.585-0.644 |
| DBP | 0.541 | 0.510-0.571 |

Abbreviation: ROC, receiver operating characteristic; END, early neurological deterioration; AUC, area under curve; CI, confidence interval; NLR, neutrophil-lymphocyte ratio; PLR. platelet-lymphocyte ratio; LMR, lymphocyte-monocyte ratio; OTT, onset to treatment time; DBP, diastolic blood pressure.
